# The RNA helicase eIF4A as a novel target in insect cells to combat arboviral infections

**DOI:** 10.1371/journal.pone.0346047

**Published:** 2026-04-06

**Authors:** Tanja Rehling, Kim Mentchen, Leonie Konopka, Wiebke Obermann, Friedemann Weber, Patrick Schmerer, Marc F. Schetelig, Arnold Grünweller, Irina Häcker, Francesca Magari

**Affiliations:** 1 Department of Insect Biotechnology in Plant Protection, Justus Liebig University Giessen, Giessen, Germany; 2 Liebig Centre for Agroecology and Climate Impact Research, International Atomic Energy Agency Collaborating Centre, Justus Liebig University Giessen, Giessen, Germany; 3 Institute of Pharmaceutical Chemistry, Philipps University Marburg, Marburg, Germany; 4 Institute for Virology, FB10-Veterinary Medicine, Justus Liebig University Giessen, Giessen, Germany; University of Wisconsin-Milwaukee, UNITED STATES OF AMERICA

## Abstract

Arthropod-borne viruses cause major global health burdens, yet few vaccines or antivirals exist. Targeting host factors required for viral replication offers a promising approach. The DEAD-box RNA helicase eIF4A, a core component of the translation initiation complex eIF4F, unwinds structured 5′ UTRs and is therefore critical also for the translation of many viral RNAs. The compound classes rocaglates and pateamines are potent eIF4A inhibitors in mammalian cells. Here we show that the natural rocaglate silvestrol strongly inhibits Rift Valley fever virus replication (RVFV) in human cells without cytotoxicity, expanding the list of eIF4A-dependent arboviruses. Moreover, we studied eIF4A function and rocaglate/pateamine sensitivity in insects, specifically in the arboviral vector *Aedes aegypti* and the fruit flies *Anastrepha suspensa* and *Drosophila melanogaster*. Sequence analysis showed conservation of the rocaglate-binding motif between the human eIF4A and all three insects. Dual luciferase assays in insect cell lines confirmed selective translation inhibition from purine-rich reporters by silvestrol below cytotoxic thresholds. Purified eIF4A variants from all three insect species retained helicase activity, allowing direct testing of inhibitor interactions. Thermal shift assays demonstrated robust stabilization of eIF4A–RNA complexes by both compound classes in the wildtype proteins, with unexpected rocaglate sensitivity of the putatively insensitive *Ae. aegypti* H161L mutant, indicating a unique binding pocket geometry of the mosquito protein. In summary, our results present RVFV as another drug target for eIF4A inhibitors and highlight comparative biochemistry studies providing insights into distinctive eIF4A inhibitor binding site architecture, with the prospect of exploring informed design to develop species-specific inhibitors.

## Introduction

Arthropod-borne viruses (arboviruses) transmitted by mosquitoes, ticks, and sandflies cause substantial global morbidity. Major mosquito-borne diseases like Zika, Dengue, Chikungunya, Rift Valley fever, West Nile, and Yellow fever are frequently vectored by *Culex* and *Aedes* spp. [1]. Disease reduction can target the vector or the virus. Current vector control relies heavily on insecticides, while vaccines and antivirals remain limited. Thus, sustainable vector control and broad-spectrum antivirals are urgently needed.

Targeting host factors essential for viral replication can provide pan-antiviral activity. A key host factor is the eukaryotic translation initiation factor 4A (eIF4A), a DEAD-box RNA helicase within the eIF4F complex that resolves structured 5′ UTRs to enable 43S preinitiation complex loading [2]. Many viral RNAs contain structured 5′ UTRs and depend on the unwinding activity of eIF4A, making eIF4A an attractive pan-antiviral target [3–10]. eIF4A inhibitors have shown activity against diverse human pathogens, including several arboviruses, *in vitro*, *ex vivo*, and *in vivo* [11–14], and therefore could also be interesting candidates to block viral replication in the mosquito instead of the human host, thereby avoiding potential toxicity in humans [15,16].

Rocaglates and pateamines are two natural compound classes (S1 Fig in S2 File) that inhibit eIF4A via RNA-clamping: They stabilize eIF4A on purine-rich sequences and block unwinding, preventing 43S ribosomal subunit recruitment [17,18]. Structural work with human eIF4A, RocA, and an (AG)₅ RNA revealed π–π stacking between RocA and the amino acid F163 and purines A7/ G8, and hydrogen bonds involving amino acid Q195 and nucleotide G8 (S2a Fig in S2 File) [9,19]. Silvestrol’s dioxanyloxy moiety (S1 Fig in S2 File) additionally engages an arginine-rich pocket critical for complex formation (S2b Fig in S2 File) [14] and can broaden sequence tolerance via interaction with a third nucleotide (A9) [20]. Amino acid position 163 (human protein numbering) is key for rocaglate sensitivity. Studies of wild type eIF4A variants in a wide variety of organisms have shown that the amino acids F/Y/H at the respective position support rocaglate binding, whereas the amino acids L/I/G/S confer rocaglate insensitivity [14,21]. In contrast, pateamine-mediated clamping is largely independent of the amino acid at this position [20,21].

So far, we and others have shown broad and potent antiviral activity of eIF4A inhibitors against a large set of highly pathogenic viruses such as Ebola-, Lassa-, Crimean Congo hemorrhagic fever-, and coronavirus, as well as the arboviruses Zika and Chikungunya *in vitro*, *ex vivo*, and also *in vivo* [3–6,8,10]. Here we extend eIF4A inhibitor activity to Rift Valley fever virus (RVFV) and test whether rocaglates and pateamines inhibit translation also in insect cells. We compare eIF4A sensitivity in the arboviral vector *Aedes aegypti* and the fruit flies *Anastrepha suspensa* and *Drosophila melanogaster* using cell-based assays and purified wild-type and mutant proteins. We show robust eIF4A–RNA clamping by both inhibitor classes and species-specific differences in rocaglate sensitivity likely due to binding pocket variation, which could be further explored for the potential to design insect-specific eIF4A inhibitors.

## Results

### Silvestrol inhibits RVFV replication

**Fig 1:**
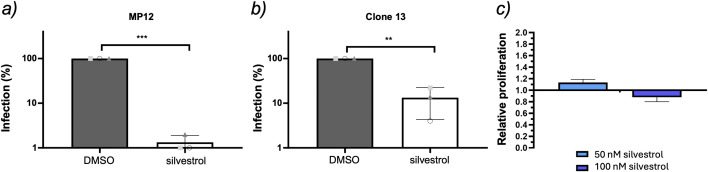
Effect of silvestrol on RVFV. RVFV L-segment RNA levels in A549 cells infected with RVFV strains MP-12 (a) or Clone 13 (b) after pretreatment with 50 nM silvestrol; (c) WST-1 cell proliferation assay in A549 cells (50–100 nM) to assess silvestrol cytotoxicity; * p ≤ 0.05; ** p ≤ 0.01 and *** p ≤ 0.001.

Rocaglate sensitivity has been shown for multiple arboviruses [9]. Here, we tested RVFV (family *Phenuiviridae*, order *Bunyavirales*) using two strains (MP-12 and Clone 13). A549 cells pretreated with 50 nM silvestrol and infected at MOI 0.1 showed a strong reduction of RVFV L-segment RNA levels for both strains relative to DMSO controls (Fig 1a, b). No cytotoxicity of silvestrol was detected at 50–100 nM (Fig 1c). Thus, in agreement with a previous study [22], these data indicate that RVFV replication is sensitive to eIF4A inhibition. The results are also consistent with the notion that RVFV protein synthesis is dependent on the eIF4A-containing eIF4F translation initiation complex [23].

### Conservation of amino acids in the eIF4A RNA binding pocket between human and insects

Instead of or in addition to targeting arboviruses by rocaglates or pateamines, the vector itself could also be a target. To estimate if known human eIF4A inhibitors may also have an effect on one of the main arboviral vectors, the similarity between eIF4A from the yellow fever mosquito *Ae. aegypti* (*Culicidae*) and human eIF4A was analyzed. In addition, we were interested in the sensitivity of other insect families from the order of *Diptera* and chose *A. suspensa* as a representative for the *Tephritidae* and *D. melanogaster* for the *Drosophilidae.*

**Fig 2:**
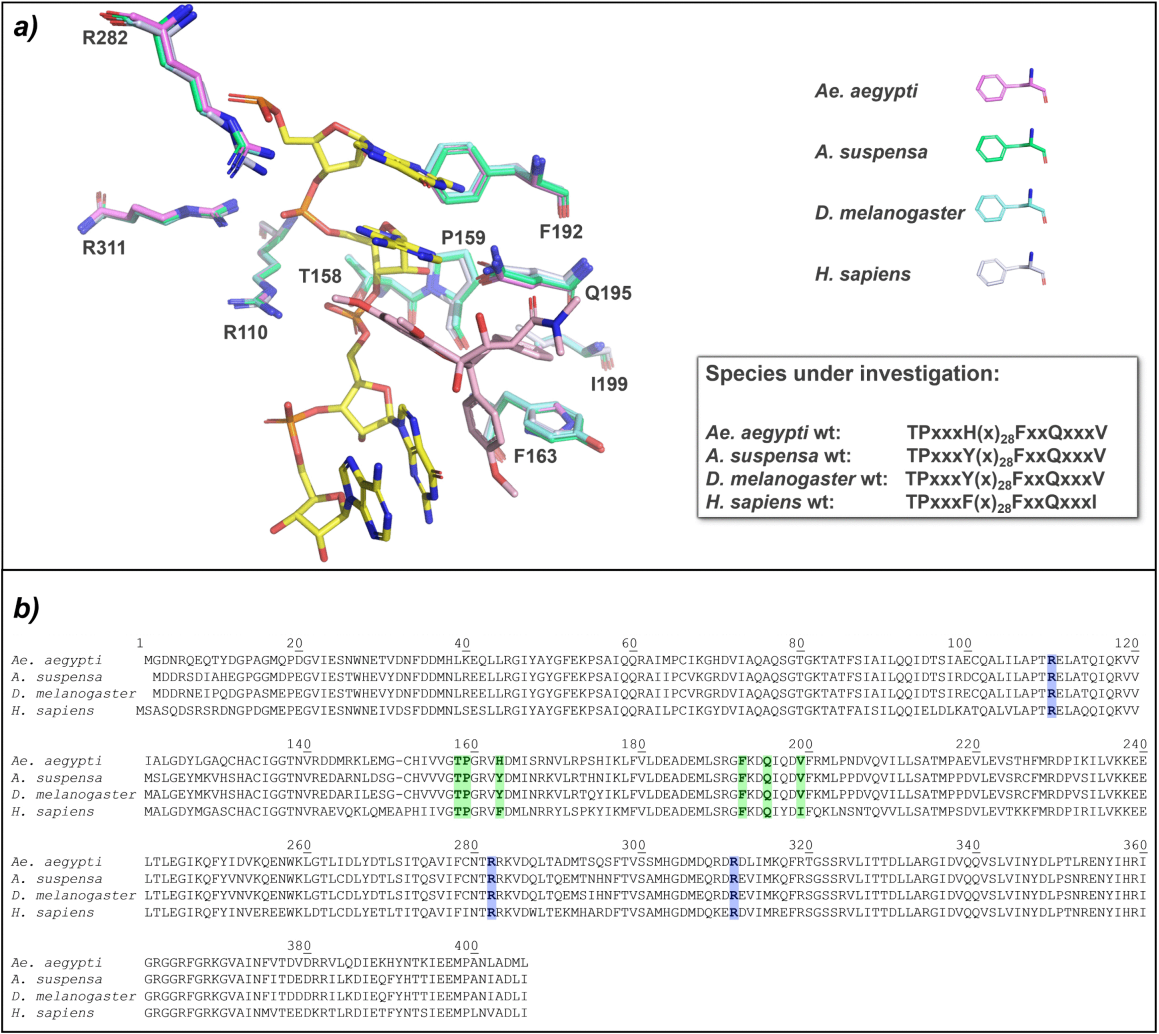
Comparison of the amino acid pattern in the eIF4A-RNA binding pocket between human eIF4A and the three insect species. a) Structure overlap between the RocA (pink) bound to human eIF4A (light blue) with (AG)₅ RNA (yellow) (PDB: 5ZC9), highlighting key residues (T158, P159, F163, F192, Q195, I199; human protein amino acid positions). *Ae. aegypti* eIF4A structure is shown in violet, *A. suspensa* in green, and *D. melanogaster* in cyan. The conserved six-residue binding motif across species (highlighted in green in b)) differs in human F163 and I199 position. b) Sequence alignment of the species under investigation. Human F163 corresponds to H161 in *Ae. aegypti* and Y160 in *A. suspensa* and *D. melanogaster*. Mutations introducing L at this position model rocaglate insensitivity. The arginine pocket is highlighted in blue.

At the molecular level, inhibition of eIF4A by RNA-clamping is mediated by interactions of rocaglates with at least two purines in the bound RNA substrate and π-π stacking interactions with a phenylalanine (F163) as part of a six amino acid motif in the rocaglate binding pocket of the human eIF4A. Comparison of the amino acid sequence in the RNA binding pocket of eIF4A showed that the six amino acid motif is highly conserved between *Homo sapiens* and the insect species under investigation (Fig 2a). Only at position 3 of the motif, corresponding to human F163, variation was observed. However, the amino acid substitutions found in the insect proteins (H or Y) have been shown to preserve RNA-clamping ability of rocaglates [14]. We, therefore, predicted that a polypurine sequence should be clamped on the surface of eIF4A from these organisms. Instead, a leucine, isoleucine, serine, or glycine at this position should prevent rocaglate binding [14,21]. Interestingly, RNA-clamping of pateamines is independent of the amino acid at position 163 in human eIF4A [21].

### Silvestrol inhibits translation initiation in insect cells

As key amino acids in the insect eIF4A RNA binding pockets indicated rocaglate sensitivity, we tested the effects of silvestrol on translation efficiency in Aag2 (*Ae. aegypti*) and S2 (*D. melanogaster*) cell lines using a modified dual luciferase reporter assay (DLA). The DLA plasmid contained a polypurine (AG)_15_ sequence serving as purine-rich 5´ UTR upstream of the firefly luciferase CDS. This should confer eIF4A dependence to firefly luciferase translation, thus allowing to test if the insect eIF4A proteins are sensitive to silvestrol. A control plasmid contained a mixed purine-pyrimidine sequence (AC)_15_, which is not dependent on eIF4A binding for translation initiation [7,19]. Firefly luciferase translation from the control plasmid should not be affected by silvestrol. The original DLA plasmids, designed for use in human cells [6], were adapted for use in insect cells by exchanging the promoter and HCV IRES element (S3 Fig in S2 File).

**Fig 3:**
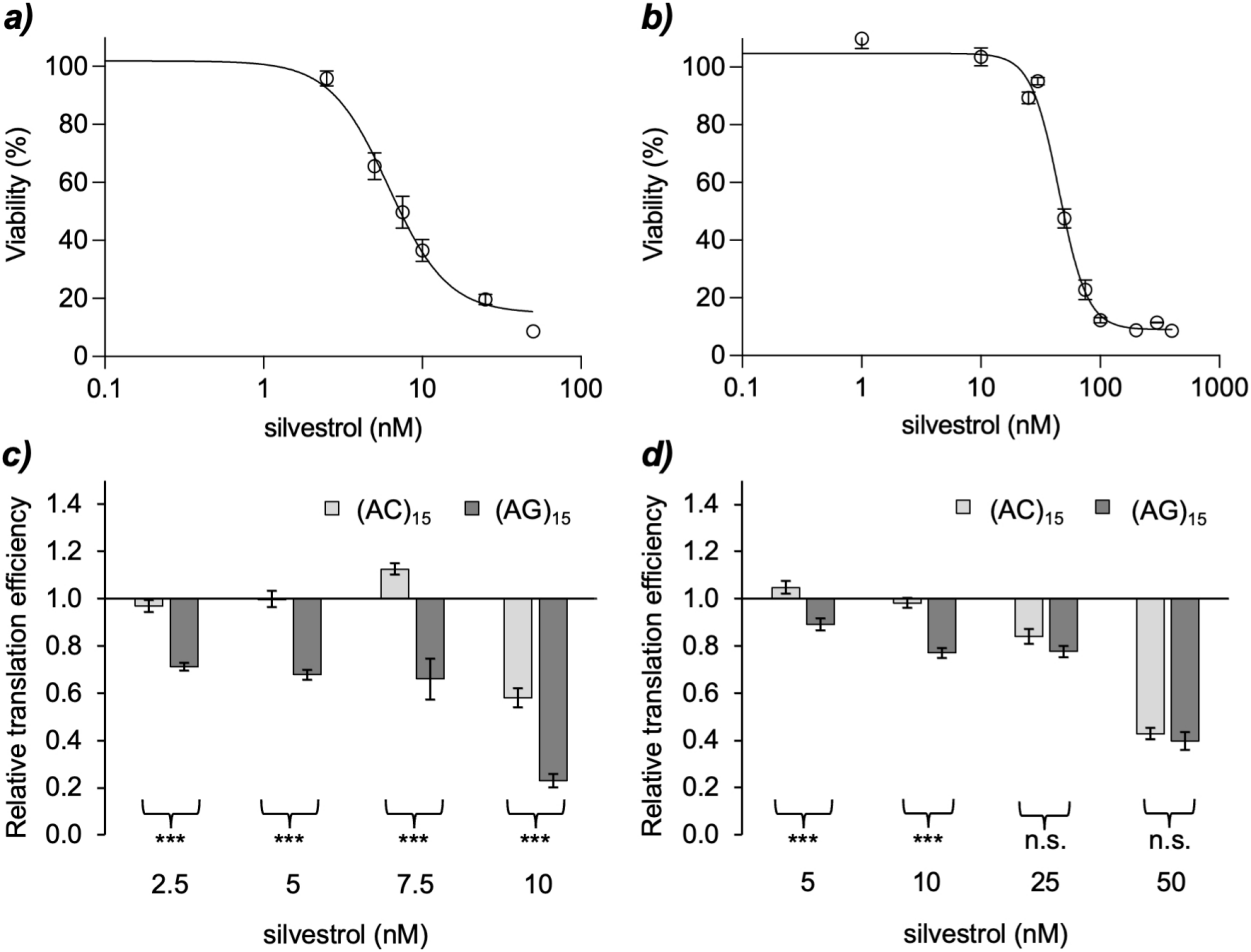
Effect of silvestrol in insect cell lines. a), b) CC_50_ value determination for silvestrol cytotoxicity in a) *D. melanogaster* S2 cells and **b)** *Ae. aegypti* Aag2 cells. Incubation time in the presence of silvestrol was 48 h. Data shown are based on a) 3-5 replicates in two independent experiments, except for 50 nM, which was only determined once, b) 3-7 replicates in 1-2 independent experiments, except for 400 nM, which was only determined once. R2 (S2) = 0.9415, R2 (Aag2) = 0.9764; c), d) Effect of silvestrol on translation efficiency determined by DLA in c) *D*. *melanogaster* S2 cells (t = 24 h) and d) *Ae*. *aegypti* Aag2 cells (t = 48 h). Interaction of silvestrol with insect eIF4A was tested with a polypurine (AG)₁₅ in the 5′ UTR of the reporter construct. The same construct with a mixed purine-pyrimidine (AC)₁₅ sequence served as silvestrol-insensitive control. Shown are means of the relative translation efficiency (firefly activity normalized to renilla activity and to the corresponding DMSO controls). Data shown are based on 2-3 independent experiments with 3 replicates each; error bars represent SEM; * p ≤ 0.05; ** p ≤ 0.01; *** p ≤ 0.001.

To exclude that the reduction in firefly luciferase expression in the DLA is due to cytotoxic silvestrol concentrations instead of RNA-clamping by silvestrol, cytotoxicity was first re-evaluated for Aag2 and S2 cells using the WST-1 cell proliferation assay. Different sensitivities to silvestrol were observed: while the CC_50_ of the *Aedes* Aag2 cell line was 55.6 nM after 24 h incubation and 49 nM after 48 h, *Drosophila* S2 cells exhibited higher sensitivity, with a CC_50_ value of 11.5 nM after 24 h of incubation and 7.3 nM after 48 h (Fig 3a, b, S4 Fig in S2 File). Based on these results, silvestrol concentrations were titrated in the DLA up to the CC_50_ values.

For both cell lines, silvestrol concentrations in the sub-CC_50_ range resulted in a strong reduction in firefly luciferase activity with the (AG)_15_ plasmid compared to the DMSO controls and to the (AC)_15_ control plasmid (Fig 3c, d), indicating efficient polypurine-dependent RNA-clamping by silvestrol onto eIF4A. Silvestrol concentrations in the CC_50_ range led to a reduction of translation efficiency also with the (AC)_15_ control in S2 cells, confirming the cytotoxicity results of the cell proliferation assays. In Aag2 cells, this effect was already visible at the half CC_50_ concentration (Fig 3d), potentially reflecting added stress to the cells due to the transfection, or an increased silvestrol uptake mediated by the transfection reagent.

### Overexpressed insect eIF4A variants show unwinding activity

**Fig 4:**
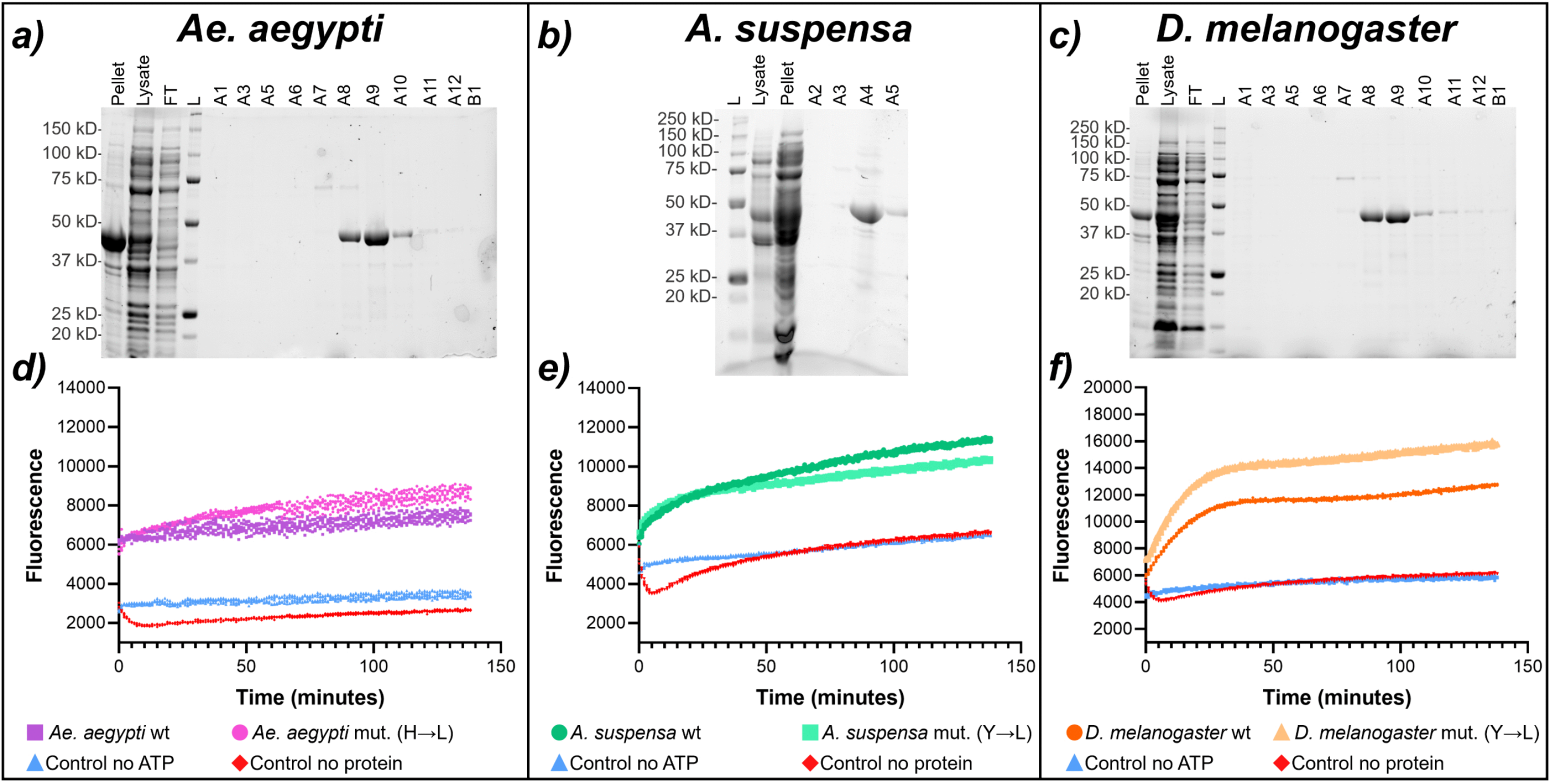
Protein purification and helicase assays. a) – c) TGX stain-free PAGE images showing successful protein expression and purification of eIF4A wt from a) *Ae*. *aegypti* (MW = 47.6 kDa), b) *A*. *suspensa* (MW = 46.0 kDa) and c) *D*. *melanogaster* (MW = 48.0 kDa). From left to right, the cell pellet, lysate, flow-through (FT), the leader Precision Plus Protein^TM^ Unstained Protein Standards (10 ‑ 250 kDa) (L), and the elution fractions (A) are shown; collected were fractions A8-10 in a), A4-5 in b), and A8-10 in c). d) – f) helicase assays showing the increase in fluorescence over time through eIF4A-mediated unwinding of quenched, fluorescently labelled RNA substrates; d) *Ae. aegypti* (wt curve in purple, mutant H161L in pink), e) *A. suspensa* (wt curve in dark green, mutant Y160L in light green), and f) *D. melanogaster* (wt curve in dark orange, mutant Y160L in pale orange). Reactions without ATP (light blue) and without protein (red) served as negative controls. Data shown are based on at least three replicate experiments.

After demonstrating that silvestrol reduces translation in insect cells, we speculated that mutants with a leucine instead of a histidine or tyrosine at position 161 (*Ae. aegypti*) or 160 (*A. suspensa*, *D. melanogaster*), respectively, (Fig 2) should not be capable of rocaglate-mediated RNA-clamping, while pateamines should have the potential to clamp RNA independently of the amino acid at this position. To prove our assumption, we overexpressed and purified the insect eIF4A wt and mutant protein variants (Fig 4a-c) and first tested the functionality of the purified proteins in a helicase assay [21], where the unwinding over time of a fluorescently labeled but quenched RNA duplex by the purified eIF4A variants is monitored via the increase of fluorescence upon duplex separation. As shown in Fig 4d-f, all purified eIF4A enzymes showed unwinding activity, demonstrating that the expressed wt as well as mutant versions of eIF4A were functional. The higher helicase activity of the mutant proteins might be caused by the shorter storage time after purification compared to the wt proteins.

### Interaction of purified insect eIF4A with rocaglates and pateamines

**Fig 5:**
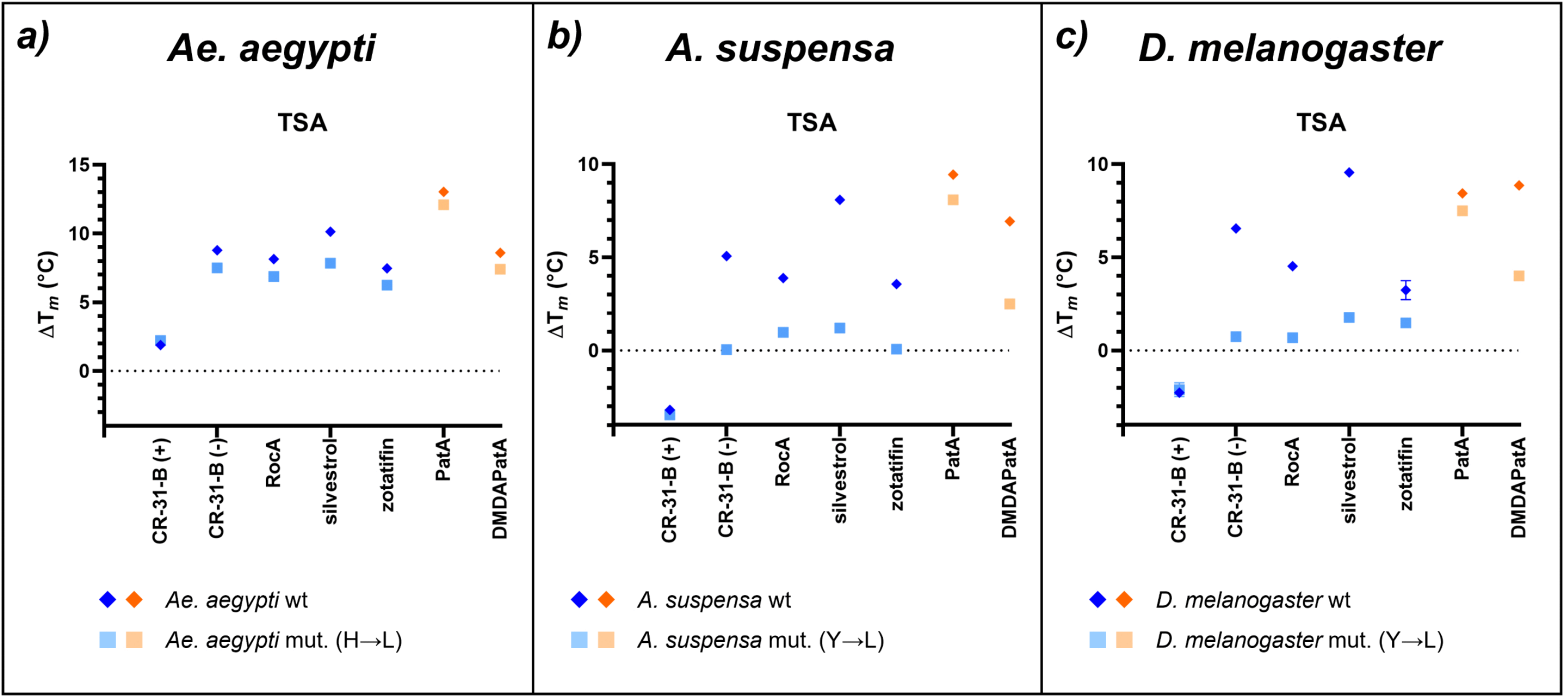
Effect of different rocaglates and pateamines on eIF4A-polypurine complex stability with wt and mutant protein versions. TSA of eIF4A from a) *Ae. aegypti*, b) *A. suspensa*, and c) *D. melanogaster* to evaluate the clamping of rocaglates (blue) and pateamines (orange) to the eIF4A-RNA complex. The wt proteins are shown in dark blue/orange rhombuses and the mutants (mut.) in light blue/orange squares. The difference in melting temperature ΔT_m_ (°C) was calculated between eIF4A and the eIF4A-(AG)_5_-AMP-PNP complex with different inhibitors. Data shown are based on at least three replicate experiments. ΔT_m_ (°C) with the corresponding standard error of the mean (SEM) for n ≥ 3 is shown (see also S2 Table in S2 File).

Next, we tested in thermal shift assays (TSA) if the purified eIF4A variants in complex with a polypurine sequence (AG)_5_ can interact with rocaglates (CR-31-B (-), RocA, silvestrol, zotatifin) or pateamines (PatA, desmethyl-desamino PatA). In TSA, the stability of proteins or protein complexes upon increase of temperature is measured with a fluorescent dye that binds to hydrophobic residues that are typically hidden in the core of the folded protein and become accessible only upon unwinding, allowing recording of a melt peak. A shift of the melt peak towards higher temperatures indicates protein (complex) stabilization, a shift to lower temperatures a destabilization. As a negative control, we used the non-functional enantiomer of the rocglate CR-31-B (-), namely, CR-31-B (+) [7], which should not stabilize the eIF4A-RNA complex. As expected, both compound classes strongly increased the thermal stability of wt eIF4A-(AG)_5_ complexes by mostly 5–10 °C (Fig 5a-c). The overall strongest effect was observed for PatA with the *Ae. aegypti* wt protein with more than 13 °C increase (Fig 5a), whereas the weakest effect was observed for zotatifin with *D. melanogaster* eIF4A showing only an increase of 3 °C in thermal stability (Fig 5c), which generally is not considered a significant shift of ΔT_m_ [14].

**Fig 6:**
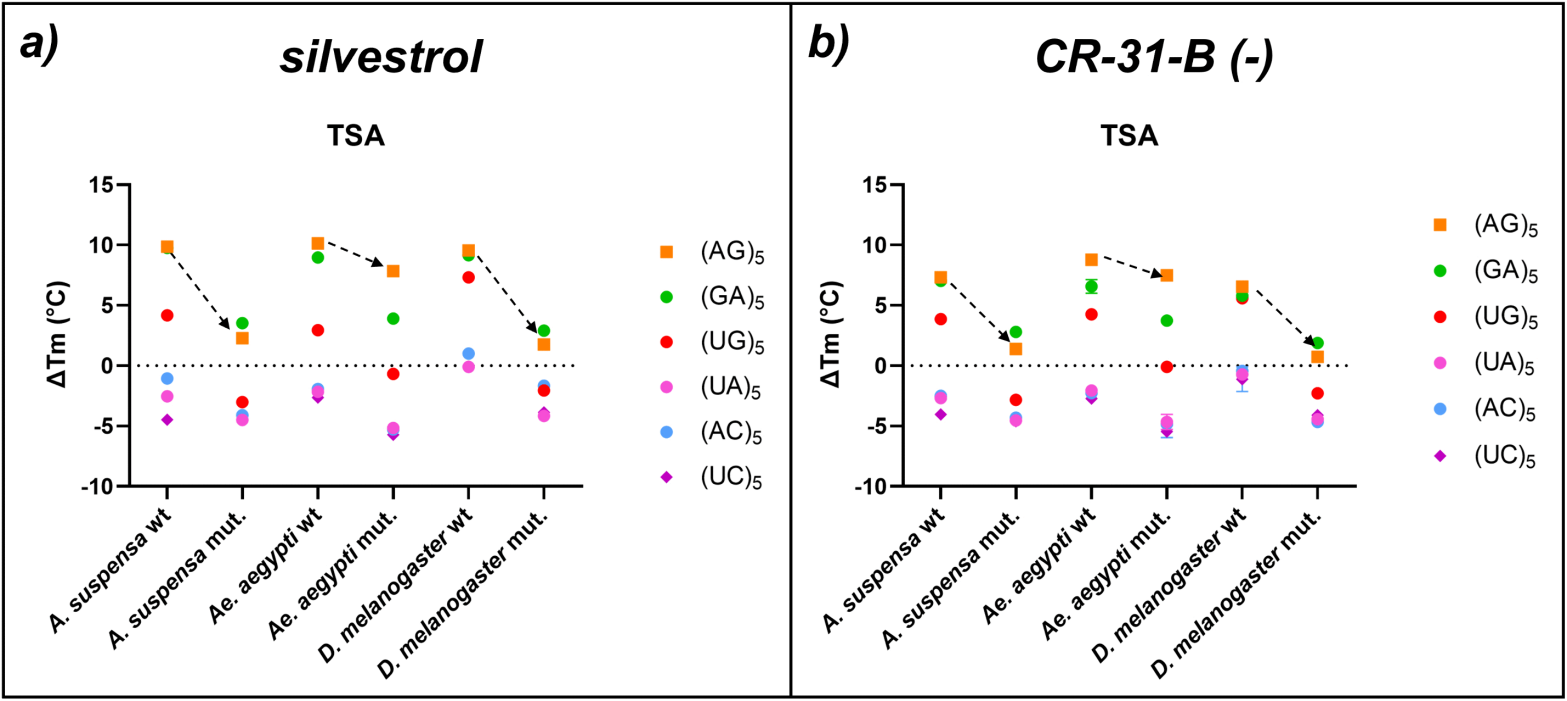
Effect of different RNA 10mers on rocaglate binding to eIF4A wt and mutant proteins. The rocaglates silvestrol (a) and CR-31-B (-) (b) were tested on polypurine stretches ((AG)_5_, orange and (GA)_5,_ green), a polypyrimidine stretch ((UC)_5_, violette) and mixed purine-pyrimidine stretches ((UG)_5_, red, (UA)_5_, pink, and (AC)_5_, light blue) in combination with *A. suspensa*, *Ae. aegypti*, and *D. melanogaster* wt as well as the mutant (mut.) eIF4A versions. Data shown are based on at least three replicate experiments. The difference in melting temperature ΔT_m_ (°C) was calculated between the eIF4A and the eIF4A-RNA-AMP-PNP complex with the two inhibitors. ΔT_m_ (°C) with the corresponding standard error of the mean (SEM) for n ≥ 3 is shown (see also S3 Table in S2 File).

Interestingly, silvestrol with its additional dioxan moiety has the strongest thermal stabilization effect in all three insect eIF4A variants compared to the other rocaglates that lack the dioxan ring. As predicted, the mutant versions of eIF4A from *A. suspensa* and *D. melanogaster* showed no significant increase in thermal stability in the presence of rocaglates, indicating that these molecules cannot clamp the RNA when a leucine is present instead of tyrosine. Unexpectedly, the H to L mutant of *Ae. aegypti* showed a similar increase in thermal stability as the wt protein (Fig 6a, light and dark blue), indicating that in this mosquito the architecture of the rocaglate binding pocket must be different (see Discussion).

For the pateamines, PatA was able to clamp (AG)_5_ also in the mutants as expected (Fig 5a-c, red and orange) and showed a stronger clamping effect with *Aedes* and *Anastrepha* proteins than the rocaglates. DMDAPatA, however, behaved differently. It showed a weaker thermal stabilization effect than PatA in *Ae. aegypti* and *A. suspensa* (Fig 5a, b). Moreover, there was a strong difference in thermal stability between wt and mutant protein in *A. suspensa* and *D. melanogaster* (Fig 5b, c).

### Different complex stabilization for polypurines, purine-pyrimidine mixes, and polypyrimidines

In a next set of TSA experiments we tested which RNA substrates can form a complex with the eIF4A wt and mutant proteins (Fig 6). Thermal stabilization was analyzed in the presence of silvestrol or CR-31-B (-) with different RNA-10mers as substrates. Silvestrol and CR-31-B (-) were selected due to their structure difference. The additional dioxane moiety of silvestrol may broaden the interactions with different RNA oligos. Substrates consisted of polypurines ((AG)_5_, (GA)_5_), mixed purine-pyrimidine sequences ((UG)_5_, (UA)_5_, (AC)_5_) or a polypyrimidine sequence (UC)_5_. In the presence of polypurines, we observed the expected increase in TSA for both compounds when eIF4A wt was used. The sequence (UG)_5_ produced a reduced thermal shift for *A. suspensa* and *Ae. aegypti*. However, in *D. melanogaster* the shift was comparable to that of polypurines. Changing the sequences to (UA)_5_, (AC)_5_ or to the polypyrimidine prevented thermal stabilization for all species (Fig 6a, b). The leucine mutants of *A. suspensa* and *D. melanogaster* resulted in a strong decrease in thermal stabilization (dashed arrows in Fig 6a, b), confirming the results from above. The *Ae. aegypti* mutant behaved again differently: in combination with (AG)_5_ there was no reduction of the thermal shift as observed before, whereas the (GA)_5_ 10mer showed a moderate but measurable reduction in thermal shift of about 5 °C with silvestrol and 3 °C with CR-31-B (-) with the mutant compared to the wt protein (Fig 6a, b). Again, this indicates that the mode of action of rocaglates in *Ae. aegypti* differs from other organisms in an unexpected manner. Overall, no substantial differences between silvestrol and the synthetic rocaglate CR-31-B (-) could be observed.

## Discussion

The main goal of this study was to investigate whether known eIF4A inhibitors can be used to target translation efficiency in insect cells as a potential new strategy to block transmission of arboviruses and to compare the inhibitor sensitivity of an arboviral vector with insects from other families. In this study, we found for the first time that RVFV requires eIF4A for protein synthesis, and that eIF4As from three different insect families are sensitive to rocaglate and pateamine treatment.

By clamping eIF4A to selected mRNAs, the rocaglates and pateamines negatively affect translation. A standard assay for measuring translation efficiency in human cancer cell lines is the dual luciferase reporter assay. By introducing a short polypurine sequence (AG)_15_ upstream of the firefly luciferase start codon, the effect of inhibitors on eIF4A-dependent translation can be investigated. Interestingly, the initial use of DLA plasmids designed for human cancer cells showed that neither the HSK promoter nor the HCV IRES element is functional in the insect cell lines used in this study. The reduction of the translation efficiency with the (AG)_15_ -containing 5´ UTR but not with the purine-pyrimidine control (AC)_15_ in the presence of silvestrol indicated RNA-clamping by rocaglates onto the insect eIF4A’s with 5´ UTRs that contain polypurine stretches. The observation that the faster proliferating S2 cells were more sensitive to silvestrol than the Aag2 cells matched observations from human cell culture and primary cancer cells [24]. While the S2 cells were more sensitive to silvestrol, no cytotoxicity was observed below the CC_50_ in DLA, in contrast to Aag2 cells, which showed signs of unspecific cytotoxicity already at the half CC_50_ for silvestrol, possibly due to added stress to the cells by the transfection reagent or an increased cellular silvestrol uptake mediated by the reagent. Notably, the CC_50_ values for silvestrol in Aag2 and S2 cells determined here deviate approximately by a factor of three from those in a previous study with these cell lines [14]. This might be due, firstly, to a difference in silvestrol storage conditions (frozen, in water in the previous study, which might have affected the activity of the compound [25], versus frozen, in DMSO in the current study). Additionally, cell seeding densities in the previous set of experiments were three- to four-fold higher, causing cells to reach a growth pleateau at the time of proliferation measurement. This could have resulted in reduced cell metabolism and thereby reduced silvestrol uptake and sensitivity.

**Fig 7:**
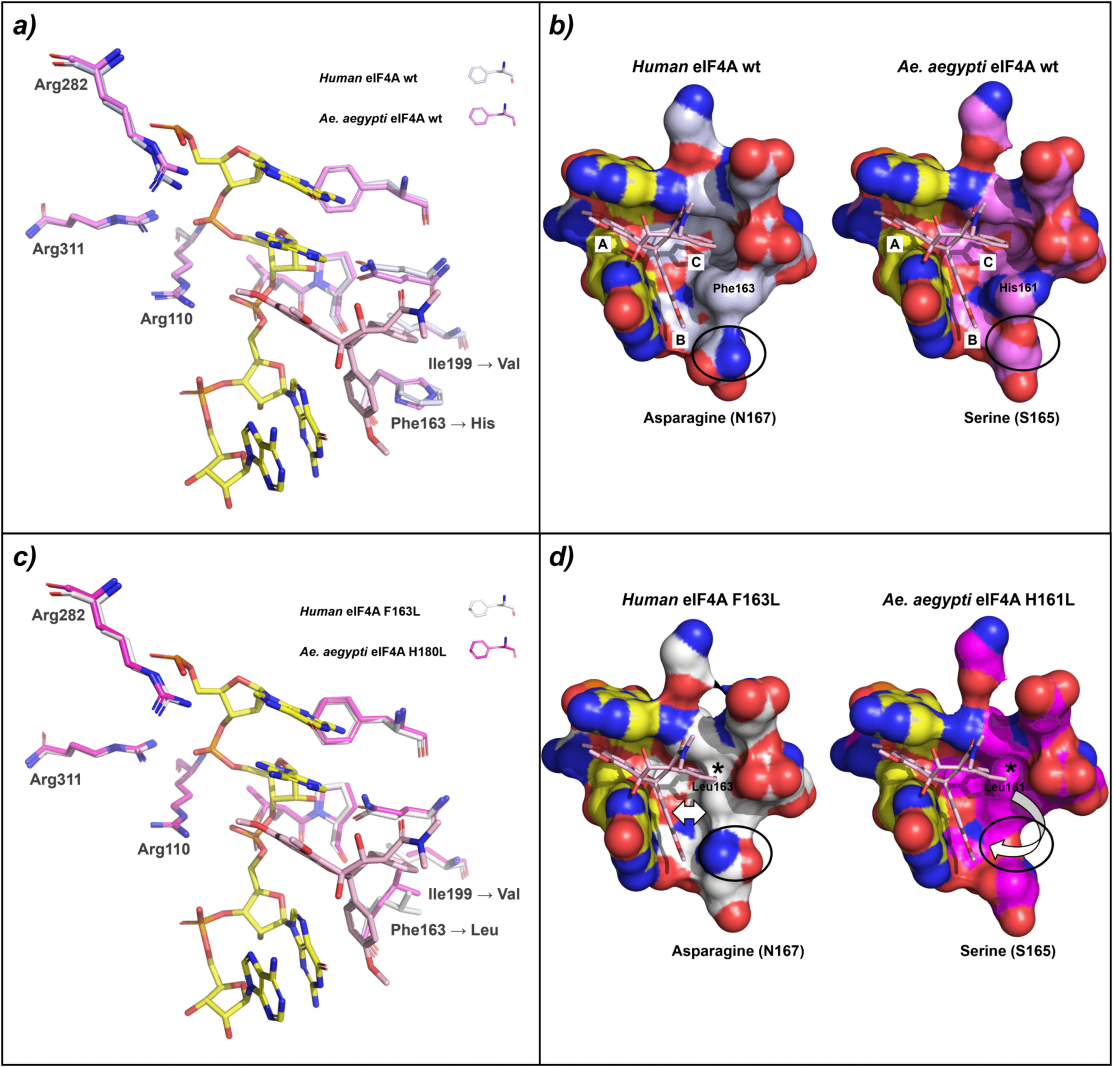
Alphafold3 structure comparison between a-b) human (silver) and *Ae. aegypti* (violet) eIF4A wt and c-d) human eIF4A H163L (white) and *Ae. aegypti* H161L (magenta) mutant proteins in complex with the polypurine (AG)_5_ RNA and the inhibitor RocA. a) Typical amino acid pattern conservation in the eIF4A-RNA binding pocket as shown in Fig 2 represented as stick model. The binding pockets of the human and *Ae. aegypti* wt eIF4A perfectly overlap. H161 in *Ae. aegypti* corresponding to F163 in human eIF4A preserves the typical π-π stacking interactions with RocA rings B and C (see S2a Fig in S2 File for more details), allowing the rocaglate to perfectly accommodate in the RNA binding pocket; b) Surface representation of a), highlighting the sub-pockets A, B and C as well as the key residues H161 and S165 in *Ae. aegypti* corresponding to F163 and N167 in human eIF4A, with RocA depicted as stick model; c) F163 in human eIF4A corresponding to H161 in *Ae. aegypti* has been mutated with a leucine residue and is represented here as a stick model. Beside a different orientation of the leucine’s side chains, the other amino acids perfectly overlap as in case of a); d) Surface representation of the RNA binding pocket highlighting the clashes between ring C of RocA and the leucine residues (L163 in human and L161 in *Ae. aegypti*). In the human eIF4A an asparagine residue (N167) closes the RNA pocket whereas in the *Ae. aegypti* the corresponding serine residue (S165) creates a wider pocket. This may promote a conformational rearrangement and better accommodation of RocA in the RNA binding pocket of *Ae. aegypti* compared to the one of the human eIF4A. The combination of the clash with L163 and a smaller pocket may prevent RocA binding to the human eIF4A F163L-RNA complex. Contrarily, the clash with L161 of *Ae. aegypti* does not hamper the binding of RocA due to a higher freedom of movement in the broader RNA binding pocket.

Overall, the mode of action of insect eIF4As compared to human eIF4A seems to be similar. Thermal shift assays with purified insect eIF4A wt and mutant proteins were used to prove the direct interaction of rocaglates or pateamines with eIF4A. The eIF4A wt and mutant proteins from *A. suspensa* and *D. melanogaster*, as well as the *Ae. aegypti* wt protein behaved similarly to the human eIF4A with respect to rocaglate-sensitivity or insensitivity. The *Ae. aegypti* eIF4A mutant, however, showed an unexpected behavior. The H161L mutant was expected to be rocaglates-insensitive, because the leucine at this position (corresponding to human F163) should prevent clamping due to the loss of π-π stacking interactions between the aromatic ring of H161 and rocaglate rings B and C (S2 Fig in S2 File). Surprisingly, the *Ae. aegypti* H161L mutant still has the ability for RNA-clamping by rocaglates (Fig 5a). This may be due to the different chemical and spatial properties of the amino acid residues surrounding the RNA binding pocket (Fig 7). On the protein surface adjacent to the key amino acid F163 (H161 or Y160 in the studied insects) an asparagine residue is present in the human eIF4A protein (human N167) as well as in the ones from *A. suspensa* and *D. melanogaster* (N164), whereas *Ae. aegypti* has a serine residue (S165) at this position (Figs 2b and 7b). Human N167 seems to close the rocaglate binding pocket, resulting in a narrower and deeper pocket with a more defined shape compared to that of *Ae. aegypti*. When F163 is mutated to L in the human eIF4A, the rocaglate ring C would clash with the latter (Fig 7d), preventing the binding and the accommodation of the inhibitor in the binding pocket, probably also due to the closure of the pocket by N167. Accordingly, an L161 in *Ae. aegypti* clashes in the model with ring C of rocaglates. Binding may not be prevented in this case because the inhibitor has more space to accommodate, possibly due to the broader pocket created by S165. This may result in a conformational rearrangement of rocaglates in the RNA binding pocket of *Ae. aegypti* that may not be possible in the human eIF4A due to N167, or in *A. suspensa* and *D. melanogaster* due to N164. To clarify this, the exact mode of action of rocaglate binding in *Ae. aegypti* should be addressed in a follow-up study.

## Materials and methods

### Effect of silvestrol on RVFV infections

Human A549 cells were obtained from ATCC (Rockville, MD) and were pretreated with DMSO or 50 nM silvestrol for 1 h, triple-washed, and infected with RVFV MP-12 or RVFV Clone 13 at MOI 0.1. Cells were incubated in medium containing the corresponding compound concentration. Total RNA was extracted 18 h post-infection. As a measure of RVFV replication, RVFV L-segment RNA was quantified by two-step qRT-PCR and normalized to GAPDH as described [26]. DMSO controls were set to 100%. Statistics: three independent experiments, paired two-sided t-test.

### Identification of insect eIF4A1 genes and cloning of variants for protein expression

Human eIF4A1 homologs were identified by BLAST in *D. melanogaster* (GCF_000001215.4, 2014) and *Ae. aegypti* (LVP AGWG L5.1, 2017). For *A. suspensa*, the homolog was inferred from *A. ludens* (GCF_028408465.1). The residue corresponding to human F163 is Y160 in *D. melanogaster* and *A. ludens* and H161 in *Ae. aegypti*.

Wild-type coding sequences were amplified from cDNA and cloned into pET-28a(+)_eIF4A1(19–406) [14] via Gibson Assembly using the NdeI/XhoI-opened backbone: Primers P2192/P2132 (*D. melanogaster*) and P2127/P2128 (*Ae. aegypti*). *A. suspensa* eIF4A was first amplified (P2341/P2342), TOPO-cloned and sequenced (near-identity to *A. ludens*), then re-amplified (P2386/P2132) and inserted into pET-28a(+).

Site-directed mutagenesis by PCR introduced leucine at the position corresponding to human F163: *D. melanogaster* TAC → CTT (primers P2192/P2194 and P2193/P2132), *Ae. aegypti* CAT → CTT (P2127/P2130 and P2129/P2128), *A. suspensa* TAC → CTT (P2386/P2385 and P2384/P2132). Overlapping PCR products were Gibson-assembled into NdeI/XhoI-digested pET-28a(+). All PCRs used Q5 High-Fidelity DNA Polymerase (NEB). Primer sequences are in Table S4 in S2 File.

### Adapting Dual luciferase vectors for testing the effects of silvestrol on translation in insect cells

The HSV-TK promoter of the dual luciferase plasmids pFR_HCV_xb_polyAC and pFR_HCV_xb_polyAG [8] was replaced with the hr5-ie1 enhancer/promoter [27] and the HCV IRES with the Drosophila C virus (DCV) IRES. hr5-ie1 was PCR-amplified from AH465 (pXLBacII_IE1hr5-DsRed.T3-SV40) [28] using P2108/P2109 (BglII/PstI overhangs) and ligated into BglII/PstI-digested vectors to yield V417 (polyAC) and V418 (polyAG) (Fig. S3). The DCV IRES was amplified from plasmid #707 (gift from Robert Harrell, Insect Transformation Facility, Maryland, USA) with P2317/P2318 and Gibson-cloned into SnaBI/NdeI-digested V417/V418 resulting in V428/V429. A shorter DCV IRES [29] was PCR-amplified from V429 (P2318/P2350, SpeI/NdeI) and ligated into SpeI/NdeI-digested V428/V429 to yield the final insect dual luciferase plasmids V432/V433 (S3 Fig in S2 File).

### eIF4A protein variants expression and purification

The plasmids pET-28a(+)-His_6_-eIF4A1 from *Ae. aegypti*, *A. suspensa* and *D. melanogaster* (wt and mut.) were transformed into BL21 (DE3) competent cells. Pre-cultures were grown in 100 mL of LB media with kanamycin (50 μg/mL) at 37 °C, 150 rpm for 18h, after which 5 mL were transferred to 500 mL of LB media with kanamycin (50 μg/mL) and grown until the OD_600_ reached 0.5–0.6. Expression was induced with 0.5 mM Isopropyl β-D-1-thiogalactopyranoside (IPTG) overnight at 18 °C. Cells were pelleted at 10 000 rpm for 10 min at 4 °C, resuspended in Buffer A (20 mM HEPES–NaOH [pH 7.5], 300 mM KCl, 20 mM Imidazole, 0.1 mM EDTA, 10 mM β-mercaptoethanol and 10% glycerol) and lysed by sonication (SONIFIER 250, BRANSON) in Buffer A containing lysozyme (50 µg/mL), benzonase (Benzonase® Nuclease, 5KU, Sigma-Aldrich/Merck) and one tablet of cOmplete™ Mini Protease Inhibitor Cocktail, EDTA-free (Roche. Germany). The lysates were centrifuged at 20 000 rpm for 45 min at 4 °C (Avanti™ J25. BECKMAN COULTER™) and the supernatant was collected and filtrated (Ultrafree®-MC filters, pore size 0.45 μm, Merck). The supernatants were loaded in 1 mL Ni-NTA column (HisTrap HP, GE Healthcare Life Science. Freiburg, Germany) and the column washed with 20 mL of Buffer A. Proteins eluted with 100% of imidazole gradient at a flow rate of 1 mL/min in Buffer B (20 mM HEPES–NaOH [pH 7.5], 300 mM KCl, 800 mM Imidazole, 0.1 mM EDTA, 10 mM β-mercaptoethanol and 10% glycerol). The eluted proteins were dialyzed overnight in the storage buffer (20 mM HEPES–NaOH [pH 7.5], 100 mM KCl, 5 mM MgCl_2_, 1 mM DTT and 10% glycerol) and concentrated to 2 mg/mL. The purified protein was stored at −80 °C.

### Thermal shift assays (TSA)

TSA is used to determine protein stability by measuring the melting temperature (T_m_) of a protein alone or in a complex (here with RNA oligos and inhibitors) using the fluorescent dye SYPRO Orange. The latter binds to hydrophobic residues which usually are in the core of the protein. With increasing temperature, the protein starts to unfold and expose its hydrophobic residues, allowing the dye to bind. Thus, with increasing temperature, fluorescence first increases until it reaches a peak, followed by a decrease corresponding to the dissociation of the protein-dye complex due to high temperatures. T_m_ is calculated as the inflexion point of the curve. A shift of the melting curve to higher temperature is indicative of a stabilization effect (here clamping of the protein to the RNA oligo in presence of the inhibitor), whereas a shift to lower temperature indicates a destabilization effect. Here, the difference in melting temperature ΔT_m_ (°C) was calculated between the eIF4A and the eIF4A-RNA-AMP-PNP complex with two inhibitor classes (rocaglate and pateamine in Fig 5) or different RNA oligos (Fig 6). TSA experiments were performed on a real-time PCR system (QuantStudio™ 3, Applied Biosystems, Waltham, MA, USA) in a MicroAmp™ Fast Optical 96-well plate (Applied Biosystems, Waltham, MA, USA) using QuantStudio™ Design & Analysis software (version 1.4.2.). 5 μM of recombinant eIF4A was incubated with 50 μM of a polypurine RNA (AG)_5_ (Biomers, Ulm, Germany), 1 mM AMP-PNP (Roche, Basel, Switzerland), 100 μM of inhibitors (silvestrol, RocA, CR-31-B (+), CR-31-B (-), zotatifin, PatA, DMDAPatA) and 75 μM of SYPRO Orange (S6650, Invitrogen, Carlsbad, CA, USA) in 20 mM HEPES–KOH buffer pH 7.5, 100 mM KCl, 5 mM MgCl_2_, 1 mM DTT and 10% (v/v) glycerol at RT in a final volume of 20 µL. In the first step, the protein sample was subjected to a cooling rate of 1.6 °C/s until a temperature of 10 °C was reached and kept constant for two minutes. In the second step, the temperature was increased by 0.05 °C/s until a temperature of 95 °C was reached and kept constant for one minute. In the final step, the temperature was decreased by 1.6 °C/s until 10 °C and kept constant for one minute. The wavelength of the fluorescence scan for excitation and emission was set to the spectroscopic maxima of SYPRO^®^ Orange (472 nm and 570 nm, respectively). The melting curves were analyzed using Protein Thermal Shift Software (version 1.3) from Thermo Fisher Scientific.

### eIF4A helicase assay

Helicase activities of the eIF4A variants were determined using two labelled RNA substrates: a 10mer modified with Cyanine 3 (10mer-Cy3; 5′-[CY3] GCU UUC CGGU-3′) and a 16mer modified with Black Hole Quencher2 (16mer-BHQ2; 5′-ACU AGC ACC GGA AAGC[BHQ2]-3′). An unlabeled competitor (10mer-competitor; 5′-GCU UUC CGGU-3′) was used to capture released quencher RNA. A single-stranded Cy3 RNA substrate (ssRNA) was used to determine the maximum fluorescence signal of the reaction. An aqueous solution of 1 µM of 10mer-Cy3 was mixed with 1 µM of 16mer-BHQ2 at 1:1 ratio and the reaction mix was annealed at 80 °C for 5 min to generate the dsRNA and incubated at room temperature for 1 h followed by incubation on ice for 10 min in 25 mM HEPES (pH 7.4 (KOH)). 1 µM of competitor RNA was added in 1:10 (v/v) excess to the labelled RNA substrates and the reaction was again incubated on ice for 10 min prior to adding it to the helicase reaction mix. This mix consists of 100 nM (final concentration) of ssRNA or dsRNA diluted in 100 µL of a buffer consisting of 150 mM HEPES-KOH pH 7.4, 15 mM Mg(CH3COO)2, 10 mM DTT, 500 mM CH3COOK and 1 mM ATP. eIF4A (12.5 μM final concentration) was added to the reaction and fluorescence was measured using a TECAN microplate reader (Tecan Infinite M Plex). As negative controls, reaction mixes either without protein or without ATP were included.

### Insect cell culture

Aag2 cells (*Ae. aegypti*) [30], and S2 cells (*D. melanogaster)* [31] were cultured in complete Schneider´s medium (Schneider´s Drosophila Medium (Gibco) supplemented with 10% fetal bovine serum (FBS, Merck-Sigma), 100 U/mL penicillin and 100 μg/mL streptomycin (Gibco) and 1 x MEM NEAA, Minimum Essential Medium (Gibco)) at 27 °C without CO_2_.

### Silvestrol toxicity assays

To determine the half-maximal cytotoxic concentration CC_50_ of silvestrol in the insect cell lines, proliferation assays in the presence or absence of silvestrol were carried out. Cells were seeded at a density of 3 × 10⁴ cells per well (Aag2) or 2 × 10⁴ cells per well (S2) in a transparent 96-well culture plate in complete Schneider´s medium and incubated for 24 hours at 27 °C to reach near confluency. Four wells with 100 µL of complete medium without cells served as blank. Silvestrol (stock solution 10 µM in DMSO) was diluted in complete medium immediately before start of the experiment and added to a final concentration of 0 nM – 50 nM for S2 cells and 0 nM – 400 nM for Aag2 cells. A series of identical final DMSO dilutions was used to check for DMSO toxicity. Cells were incubated at 27 °C for 24 h or 48 h, after which cell proliferation was analysed using the WST-1 assay (Sigma Aldrich) following the manufacturer’s protocol. Absorbance was measured after three hours of incubation with the WST-1 reagent at 440 nm, with 650 nm as the reference wavelength, using a Tecan SPARK reader. Absorbance for the different silvestrol concentrations was normalized to the mean value of the blank control. The CC₅₀ value of silvestrol was determined from the dose–response curves. Viability was calculated as the percentage of viable cells of the experimental groups treated with silvestrol relative to the untreated control (0 nM silvestrol/DMSO). Nonlinear regression was performed using a four-parameter logistic (4PL) model in GraphPad Prism, yielding the parameters Top, Bottom, IC₅₀, and Hill slope. Because the residual viability at high silvestrol concentrations (Bottom) did not reach zero, the CC₅₀ value was calculated by inverting the fitted 4PL function. The CC₅₀ was defined as the concentration corresponding to 50% viability and was calculated using the following equation:


$$\begin{array}{c} CC_{\text{5}\text{0}}=IC_{\text{5}\text{0}}\times {\left(\frac{Top-Bottom}{\text{5}\text{0}-Bottom}-\text{1}\right)}^{\text{1}/Hill} \end{array}$$ (1)

where Top and Bottom represent the upper and lower asymptotes of the fitted curve, respectively, and Hill denotes the Hill slope.

### Dual luciferase reporter assays

Cells were seeded 24 h before transfection (Aag2: 3 × 10⁴; S2: 2 × 10⁴ cells/well) in black clear-bottom 96-well plates. Transfection used 100 ng plasmid/well, 0.3 µL Lipofectamine 3000 reagent, and 0.3 µL P3000 reagent per well in serum-free medium according to the manufacturer’s instructions. Four hours later, medium was replaced with complete medium ± silvestrol (S2: 2.5–10 nM, 24 h; Aag2: 5–50 nM, 48 h) or DMSO at the respective dilutions as control. Firefly and Renilla luciferase activities were measured with the Dual Luciferase® Reporter Assay (Promega) using 50 µL LARII and Stop & Glo® per well on a Tecan SPARK reader. For S2, a filter was set to reduce the signal by 100× to avoid saturation. For calculation of translation efficiency of poly(AC)_15_ and poly(AG)_15_ DLA plasmids (V432 and V433, respectively) firefly luminescence was first normalized to Renilla luminescence to correct for cell number and transfection efficiency differences. Then silvestrol treatment values were normalized to the respective DMSO controls; SEMs were calculated on normalized ratios. Statistical analysis of differences in translation efficiencies of poly(AG)_15_ versus poly(AC)_15_ plasmids was performed using one-way ANOVA with Shapiro–Wilk and Brown–Forsythe tests, followed by Holm–Šidák post hoc test [32,33]. If variances were highly unequal or for unequal group sizes Kruskal–Wallis with Dunn’s test was performed [34].

### Software

For protein purification, UNICORN^TM^ (Version 6.0) was used on an ÄKTA pure system (GE Healthcare). AlphaFold Server prediction was used to predict the structure of eIF4A variants from insects (Abramson et al. 2024). The PyMOL Molecular Graphics System (Version 2.0, Schrödinger, LLC) was used to both, analyze the predicted Alphafold3 structures and for picture generation. GraphPad Prism version 9 for Windows, (GraphPad Software, Boston, Massachusetts USA; www.graphpad.com) was used for data and statistical analysis as well as to design the graphs shown in the present work. Statistical analyses of silvestrol toxicity assays and calculations of CC_50_ values of silvestrol were conducted using GraphPad Prism version 10.6.1 for macOS (GraphPad Software, Boston, Massachusetts, USA; www.graphpad.com). Statistical analysis of the dual luciferase reporter assays was performed with SigmaPlot (Systat Software GmbH).

## Supporting information

S1 File Raw images of gels shown in Fig 4. (PDF)

S2 File Supporting Information file containing S1 Table – S5 Table and S1 Fig – S4 Fig. (PDF)
