# Supplementary material for: The RNA helicase eIF4A as a novel target in insect cells to combat arboviral infections
Source: PLoS One. 2026 Apr 6;21(4):e0346047. doi: 10.1371/journal.pone.0346047 (PMC13052863; doi:10.1371/journal.pone.0346047)
Supplement: S1 File — (PDF) [file pone.0346047.s001.pdf]

a)

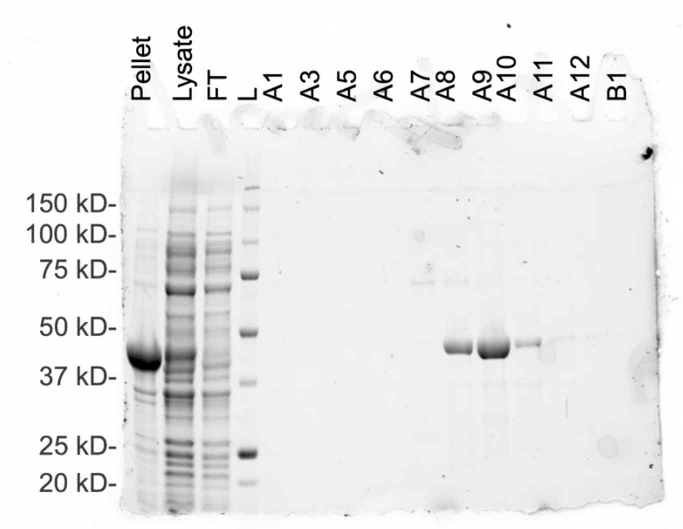

b)

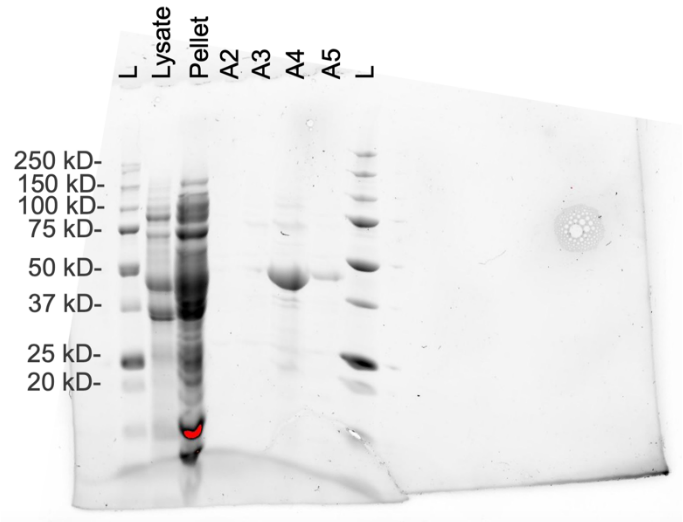

c)

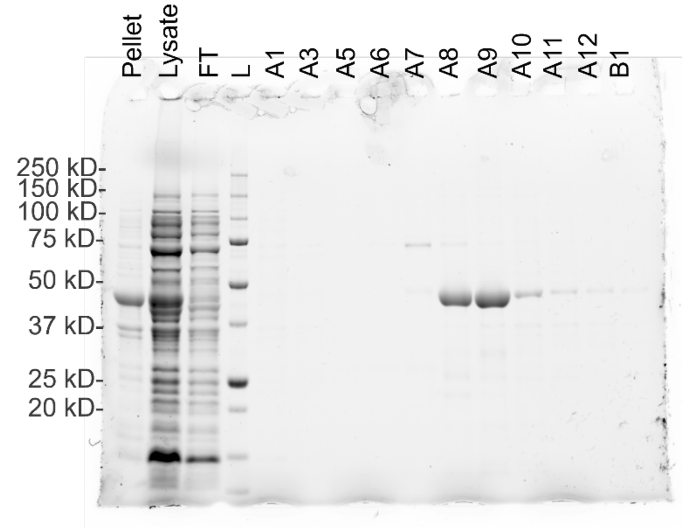

S1_raw images of gels shown in Fig 4. a) *Ae. aegypti*, b) *A. suspensa*, c) *D. melanogaster*; L = Protein Precision Plus protein standard (ladder), A = elution fractions
