# Supplementary material for: The RNA helicase eIF4A as a novel target in insect cells to combat arboviral infections
Source: PLoS One. 2026 Apr 6;21(4):e0346047. doi: 10.1371/journal.pone.0346047 (PMC13052863; doi:10.1371/journal.pone.0346047)
Supplement: S2 File — (PDF) [file pone.0346047.s002.pdf]

# Supporting Information

# The RNA helicase eIF4A as a novel target in insect cells to combat arboviral infections

Tanja Rehling[1,2*], Kim Mentchen[3*], Leonie Konopka[3], Wiebke Obermann[3], Friedemann Weber[4], Patrick Schmerer[4], Marc F. Schetelig[1,2], Arnold Grünweller[3], Irina Häcker[1,2,§], Francesca Magari[3,§]

[1] Department of Insect Biotechnology in Plant Protection, Justus Liebig University Giessen, 35394 Giessen, Germany

[2] Liebig Centre for Agroecology and Climate Impact Research, International Atomic Energy Agency Collaborating Centre, Justus Liebig University Giessen, 35394 Giessen, Germany

[3] Institute of Pharmaceutical Chemistry, Philipps University Marburg, 35032 Marburg. Germany

[4] Institute for Virology, FB10-Veterinary Medicine, Justus Liebig University Giessen, 35392 Giessen, Germany.

*equal contribution as first authors

§ corresponding authors

## Supporting Tables:

**S1 Table. Best-fit, 95% CI and Goodness-of-fit data for the non-linear regression analysis of dose-viability curves (cell proliferation rate in *D. melanogaster* S2 cells and *Ae. aegypti* Aag2 cells in presence of silvestrol).** A four-parameter logistic (4PL) model in GraphPad Prism was used.

| | S2 (24h) | S2 (48h) | Aag2 (24h) | Aag2 (48h) |
|---|---|---|---|---|
| [Inhibitor] vs. response -- Variable slope (four parameters) | | | | |
| Best-fit values | | | | |
| Bottom | 26.81 | 14.83 | 35.08 | 8.988 |
| Top | 105.7 | 101.9 | 99.47 | 104.7 |
| IC50* | 6.35 | 6.146 | 36.7 | 45.28 |
| HillSlope | -1.475 | -2.344 | -2.885 | -3.682 |
| logIC50 | 0.8028 | 0.7886 | 1.565 | 1.656 |
| Span | 78.92 | 87.04 | 64.39 | 95.74 |
| 95% CI (profile likelihood) | | | | |
| Bottom | 10,78 to 36,34 | 2,395 to 24,75 | 30,92 to 38,79 | 4,044 to 13,58 |
| Top | 102,1 to 109,4 | 95,29 to 108,8 | 92,18 to 106,8 | 101,3 to 108,2 |
| IC50 | 4,975 to 9,311 | 5,154 to 7,439 | 31,55 to 42,86 | 42,07 to 48,66 |
| HillSlope | -2,096 to -1,027 | -3,322 to -1,662 | -3,942 to -2,175 | -4,693 to -3,000 |
| logIC50 | 0,6968 to 0,9690 | 0,7121 to 0,8715 | 1,499 to 1,632 | 1,624 to 1,687 |
| Goodness of Fit | | | | |
| Degrees of Freedom | 42 | 24 | 8 | 40 |
| **R squared** | **0.9347** | **0.9415** | **0.9861** | **0.9764** |
| Sum of Squares | 2308 | 1550 | 78.61 | 1737 |
| Sy.x | 7.412 | 8.036 | 3.135 | 6.59 |
| Constraints | | | | |
| IC50 | IC50 > 0 | IC50 > 0 | IC50 > 0 | IC50 > 0 |
| Number of points | | | | |
| # of X values | 105 | 105 | 140 | 140 |
| # Y values analyzed | 46 | 28 | 12 | 44 |

**S2 Table. TSA values of eIF4A variants in complex with $(AG)_5$ RNA in presence of different rocaglates and pateamines**

| eIF4A variants | *Ae. aegypti* wt | | *Ae. aegypti* mut. | | *A. suspensa* wt | | *A. suspensa* mut. | | *D. melanogaster* wt | | *D. melanogaster* mut. | |
|---|---|---|---|---|---|---|---|---|---|---|---|---|
| **eIF4A inhibitors** | **$\Delta T_m$ (°C)** | **SEM (°C)** | **$\Delta T_m$ (°C)** | **SEM (°C)** | **$\Delta T_m$ (°C)** | **SEM (°C)** | **$\Delta T_m$ (°C)** | **SEM (°C)** | **$\Delta T_m$ (°C)** | **SEM (°C)** | **$\Delta T_m$ (°C)** | **SEM (°C)** |
| **CR-31-B (+)** | 1.89 | 0.05 | 2.21 | 0.37 | -3.20 | 0.34 | -3.46 | 0.15 | -2.27 | 0.15 | -2.10 | 0.37 |
| **CR-31-B (-)** | 8.79 | 0.08 | 7.50 | 0.06 | 5.07 | 0.15 | 0.04 | 0.10 | 6.55 | 0.00 | 0.74 | 0.10 |
| **RocA** | 8.14 | 0.05 | 6.87 | 0.10 | 3.89 | 0.26 | 0.97 | 0.29 | 4.52 | 0.26 | 0.68 | 0.20 |
| **silvestrol** | 10.14 | 0.14 | 7.84 | 0.22 | 8.09 | 0.15 | 1.20 | 0.14 | 9.55 | 0.06 | 1.76 | 0.17 |
| **zotatifin** | 7.47 | 0.15 | 6.25 | 0.29 | 3.56 | 0.09 | 0.07 | 0.00 | 3.24 | 0.51 | 1.48 | 0.06 |
| **PatA** | 13.03 | 0.03 | 12.09 | 0.03 | 9.44 | 0.09 | 8.09 | 0.18 | 8.43 | 0.03 | 7.5 | 0.05 |
| **DMDAPatA** | 8.6 | 0.05 | 7.41 | 0.05 | 6.93 | 0.15 | 2.50 | 0.06 | 8.86 | 0.05 | 4.00 | 0.05 |

**S3 Table. TSA values of eIF4A variants in complex with different RNA oligos**

| | eIF4A variants | *Ae. aegypti* wt | | *Ae. aegypti* mut. | | *A. suspensa* wt | | *A. suspensa* mut. | | *D. melanogaster* wt | | *D. melanogaster* mut. | |
|---|---|---|---|---|---|---|---|---|---|---|---|---|---|
| | **RNA oligos** | **$\Delta T_m$ (°C)** | **SEM (°C)** | **$\Delta T_m$ (°C)** | **SEM (°C)** | **$\Delta T_m$ (°C)** | **SEM (°C)** | **$\Delta T_m$ (°C)** | **SEM (°C)** | **$\Delta T_m$ (°C)** | **SEM (°C)** | **$\Delta T_m$ (°C)** | **SEM (°C)** |
| **silvestrol** | $(AG)_5$ | 10.14 | 0.14 | 7.84 | 0.22 | 9.86 | 0.15 | 2.28 | 0.14 | 9.55 | 0.06 | 1.76 | 0.17 |
| | $(GA)_5$ | 8.96 | 0.02 | 3.91 | 0.00 | 9.75 | 0.00 | 3.53 | 0.10 | 9.16 | 0.15 | 2.90 | 0.06 |
| | $(UG)_5$ | 2.95 | 0.23 | -0.68 | 0.10 | 4.18 | 0.15 | -3.02 | 0.25 | 7.31 | 0.09 | -2.05 | 0.35 |
| | $(UA)_5$ | -2.16 | 0.06 | -5.16 | 0.15 | -2.53 | 0.10 | -4.50 | 0.16 | -0.09 | 0.00 | -4.15 | 0.32 |
| | $(AC)_5$ | -1.93 | 0.06 | -5.7 | 0.10 | -1.05 | 0.06 | -4.10 | 0.16 | 1.02 | 0.09 | -1.65 | 0.17 |
| | $(UC)_5$ | -2.64 | 0.09 | -5.73 | 0.11 | -4.46 | 0.15 | -4.15 | 0.34 | -0.09 | 0.00 | -3.87 | 0.17 |
| **CR-31-B (-)** | $(AG)_5$ | 8.79 | 0.08 | 7.50 | 0.06 | 7.32 | 0.15 | 1.39 | 0.11 | 6.55 | 0.00 | 0.74 | 0.10 |
| | $(GA)_5$ | 6.58 | 0.58 | 3.74 | 0.00 | 7.04 | 0.14 | 2.81 | 0.14 | 5.81 | 0.34 | 1.88 | 0.06 |
| | $(UG)_5$ | 4.25 | 0.17 | -0.09 | 0.09 | 3.86 | 0.06 | -2.82 | 0.22 | 5.59 | 0.06 | -2.27 | 0.35 |
| | $(UA)_5$ | -2.04 | 0.10 | -4.65 | 0.63 | -2.67 | 0.12 | -4.53 | 0.05 | -0.71 | 0.06 | -4.43 | 0.15 |
| | $(AC)_5$ | -2.27 | 0.06 | -4.82 | 0.32 | -2.5 | 0.07 | -4.03 | 0.18 | -0.43 | 0.26 | -4.66 | 0.45 |
| | $(UC)_5$ | -2.73 | 0.34 | -5.44 | 0.51 | -4.03 | 0.30 | -4.59 | 0.19 | -1.11 | 1.02 | -4.09 | 0.25 |

## S4 Table. Primers

| Name | Sequence |
|---|---|
| P2108 | TCAAGATCTCGGGGGATCCGGCGCGTAA |
| P2109 | TCACTGCAGTCTTGTCGCCGCCAGTGTCAAC |
| P2132 | TGGTGGTGGTGGTGGTGCCTCGAGTTAAATCAAATCGGCAATATTAGCAGG |
| P2127 | GGTGCCGCGCGGCAGCCATATGGGCGATAACAGACAGGAG |
| P2128 | TGGTGGTGGTGGTGGTGCTCGAGTTACAGCATATCAGC |
| P2129 | ACGTGTGCTTGACATGATCAGC |
| P2130 | GCTGATCATGTCAAGCACACGTC |
| P2192 | GGTGCCGCGCGGCAGCCATATGGATGACCGAAATGAGATACCT |
| P2193 | GCCGCGTCCTTGACATGATC |
| P2194 | TCATGTCAAGGACGCGGC |
| P2317 | CATGGTGGCTAGACATATGCAAATAATCTGTTGAAAGACTAACGA |
| P2318 | CCGTGTAATTCTAGTGTACTAGTTAAGATGTGATCTTGCTTCCT |
| P2341 | ATGGATGATCGAAGTGATATAGCTCATG |
| P2342 | TTAAATCAAATCGGCAATATTAGCAGGC |
| P2350 | ATCGCATATGATTGTTTGAAAGTTAGCAGGTT |
| P2384 | GGCGTGTTCTTGACATGATT |
| P2385 | TCATGTCAAGAACACGCC |
| P2386 | GGTGCCGCGCGGCAGCCATATGGATGATCGAAGTGATATAGCTCA |

## S5 Table. Sequences

| Name | Sequence |
|---|---|
| hr5-ie1 enhancer-promotor | AGGGGGATCCGGCGCGTAAAACACAATCAAGTACGAGTCATAAGCTGATGTCATGTTTTGCACACG<br>GCTCATAACCGAACTGGCTTTACGAGTAGAATTCTACTTGTAACGCACGATCAGTGGATGATGTCAT<br>TTGTTTTTCAAATCGAGATGATGTCATGTTTTGCACACGGCTCATAAACTCGCTTTACGAGTAGAATT<br>CTACGTGTAACGCACGATCGATTGATGAGTCATTTGTTTTGCAATATGATATCATACAATATGACTCA<br>TTTGTTTTTCAAAACCGAACTTGATTTACGGGTAGAATTCTACTTGTAAAGCACAATCAAAAAGATGA<br>TGTCATTTGTTTTTCAAAACTGAACTCGCTTTACGAGTAGAATTCTACGTGTAAAACACAATCAAGAA<br>ATGATGTCATTTGTTATAAAAATAAAAGCTGATGTCATGTTTTGCACATGGCTCATAACTAAACTCGC<br>TTTACGGGTAGAATTCTACGCGCCGGATCCACTAGCTAGTTCTAGAGTCGATGTCTTTGTGATGCG<br>CGCGACATTTTTGTAGGTTATTGATAAAATGAACGGATACGTTGCCCGACATTATCATTAAATCCTTG<br>GCGTAGAATTTGTCGGGTCCATTGTCCGTGTGCGCTAGCATGCCCGTAACGGACCTCGTACTTTTG<br>GCTTCAAAGGTTTTGCGCACAGACAAAATGTGCCACACTTGCAGCTCTGCATGTGTGCGCGTTACC<br>ACAAATCCCAACGGCGCAGTGTACTTGTTGTATGCAAATAAATCTCGATAAAGGCGCGGCGCGCGA<br>ATGCAGCTGATCACGTACGCTCCTCGTGTTCCGTTCAAGGACGGTGTTATCGACCTCAGATTAATG<br>TTTATCGGCCGACTGTTTTCGTATCCGCTCACCAAACGCGTTTTTGCATTAACATTGTATGTCGGCG<br>GATGTTCTATATCTAATTTGAATAAATAAACGATAACCGCGTTGGTTTTAGAGGGCATAATAAAAGAA<br>ATATTGTTATCGTGTTCGCCATTAGGGCAGTATAAATTGACGTTCATGTTGGATATTGTTTCAGTTGC<br>AAGTTGACACTGGCGGCGACAAGA |
| $(AC)_{15}$ | ACACACACACACACACACACACACACACAC |
| $(AG)_{15}$ | AGAGAGAGAGAGAGAGAGAGAGAGAGAGAG |
| *Drosophila* C virus IRES | TAAGATGTGATCTTGCTTCCTTATACAATTTTGAGAGGTTAATAAGAAGGAAGTAGTGCTATCTTAAT<br>AATTAGGTTAACTATTTAGTTTTACTGTTCAGGATGCCTATTGGCAGCCCCATAATATCCAGGACAC<br>CCTCTCTGCTTCTTATATGATTAGGTTGTCATTTAGAATAAGAAAATAACCTGCTAACTTTCAAACAA |

| | |
|---|---|
| *D. melanogaster eIF4A1*-wt | ATGGATGACCGAAATGAGATACCTCAGGATGGCCCCGCCAGCATGGAACCCGAGGGCGTCATCGA<br>GTCCACCTGGCACGAGGTGTACGACAACTTCGATGACATGAACTTGCGCGAGGAGTTGCTGCGCG<br>GCATCTACGGTTATGGTTTCGAGAAGCCGTCGGCCATCCAGCAGCGCGCCATCATTCCCTGTGTG<br>AGGGGTCGCGATGTCATTGCCCAGGCCCAGTCGGGAACTGGCAAGACTGCCACCTTCTCGATTGC<br>TATCCTTCAGCAAATCGACACGAGCATTCGCGAGTGCCAGGCGCTGATCCTGGCCCCCACTCGCG<br>AGTTGGCCACGCAGATCCAGCGCGTGGTGATGGCGCTCGGCGAGTACATGAAGGTGCACTCGCA<br>CGCCTGCATTGGCGGCACTAACGTGCGCGAAGACGCCCGCATCTTGGAATCCGGTTGCCATGTGG<br>TGGTGGGCACTCCTGGCCGCGTCTACGACATGATCAACCGCAAGGTGCTGCGCACCCAGTACATC<br>AAGCTGTTCGTGCTGGATGAGGCCGATGAGATGTTGTCCCGCGGTTTCAAGGATCAGATCCAGGA<br>TGTCTTCAAGATGCTGCCCCCAGATGTGCAGGTCATCCTGCTGTCCGCCACCATGCCGCCGGATG<br>TGCTCGAAGTGAGCCGTTGCTTCATGCGTGATCCCGTCAGCATCCTGGTTAAGAAGGAGGAACTGA<br>CCCTTGAGGGTATCAAGCAGTTTTACGTCAACGTGAAGCAGGAGAACTGGAAACTGGGCACCCTTT<br>GCGATCTGTACGATACGCTGTCCATCACCCAGTCGGTAATCTTCTGCAACACCCGTCGCAAGGTGG<br>ACCAACTGACCCAGGAGATGTCTATCCACAACTTCACCGTCTCGGCCATGCACGGCGACATGGAG<br>CAGCGTGATCGCGAGGTCATCATGAAACAATTCCGTTCGGGCTCGTCTCGTGTTCTGATTACCACT<br>GATTTACTGGCGCGCGGTATTGATGTGCAGCAGGTGTCGTTGGTCATCAACTATGATCTGCCCTCG<br>AACCGCGAGAACTACATTCATAGAATTGGTCGCGGTGGTCGTTTCGGTCGCAAGGGTGTTGCGATC<br>AACTTTATTACAGATGATGATCGACGAATCCTTAAGGATATTGAACAGTTCTACCACACAACAATTGA<br>GGAAATGCCTGCTAATATTGCCGATTTGATTTAA |
| *Ae. aegypti eIF4A1*-wt | ATGGGCGATAACAGACAGGAGCAGACTTACGATGGACCCGCTGGTATGCAACCGGACGGAGTCAT<br>CGAGTCCAACTGGAACGAGACCGTCGACAACTTCGACGATATGCACCTGAAGGAGCAGCTGCTCC<br>GTGGCATCTACGCCTACGGTTTCGAGAAGCCGTCTGCTATCCAACAGCGCGCTATTATGCCGTGCA<br>TCAAGGGTCACGATGTGATTGCCCAGGCTCAATCTGGTACTGGTAAAACTGCTACGTTTTCCATTG<br>CGATCTTGCAGCAGATCGACACCAGCATTGCAGAATGCCAAGCGCTGATTCTGGCTCCAACCCGT<br>GAGTTGGCCACCCAGATCCAGAAGGTAGTGATCGCCCTCGGCGATTACCTGGGCGCCCAATGCCA<br>TGCCTGCATTGGAGGTACGAATGTCCGCGACGACATGCGTAAACTGGAAATGGGCTGCCACATCG<br>TGGTCGGAACCCCGGGACGTGTGCATGACATGATCAGCCGTAATGTCCTGCGCCCATCGCACATC<br>AAGCTTTTCGTCCTGGACGAAGCTGATGAAATGTTGTCCCGTGGTTTCAAGGACCAGATTCAGGAT<br>GTTTTCCGTATGTTGCCCAATGATGTACAGGTCATTCTTCTCTCCGCTACCATGCCAGCTGAAGTGT<br>TGGAAGTCTCCACCCACTTCATGCGTGACCCGATCAAGATTTTGGTCAAGAAGGAAGAACTGACAC<br>TGGAAGGTATCAAACAATTCTACATCGATGTGAAGCAGGAAAACTGGAAACTCGGCACACTAATCG<br>ATCTGTACGATACGCTATCCATCACTCAGGCCGTCATCTTCTGCAACACCCGTCGTAAGGTCGACC<br>AGCTGACCGCTGATATGACCTCCCAGAGCTTCACCGTTTCTTCCATGCACGGTGACATGGACCAGC<br>GTGATCGTGACTTGATTATGAAGCAGTTCCGTACTGGATCGTCCCGTGTCCTCATCACCACGGATC<br>TTCTCGCTCGTGGTATCGACGTGCAGCAGGTTTCCCTAGTTATTAATTATGATTTACCAACGTTAAG<br>AGAAAACTACATCCACAGAATTGGCCGAGGTGGTCGTTTCGGTCGTAAGGGAGTTGCAATCAACTT<br>CGTTACTGATGTTGACAGAAGAGTGCTGCAAGACATAGAGAAACATTACAACACCAAAATCGAAGA<br>GATGCCAGCAAATCTGGCTGATATGCTGTAA |
| *A. ludens eIF4A1*-wt | ATGGATGATCGAAGTGATATAGCTCATGAAGGTCCAGGCGGTATGGATCCTGAGGGTGTCATCGA<br>GTCAACTTGGCACGAGGTGTATGATAATTTCGATGACATGAATTTGCGCGAAGAGTTATTGCGAGG<br>TATCTATGGTTATGGTTTTGAAAAGCCTTCTGCTATTCAGCAGCGTGCTATCATTCCTTGTGTAAAG<br>GGCCGCGACGTTATTGCCCAGGCGCAGTCGGGTACGGAAAAACTGCTACTTTTTCCATTGCAATC<br>TTGCAACAAATCGATACGTCCATCCGTGACTGTCAAGCGTTGATTTTGGCCCCAACTAGGGAATTG<br>GCAACCCAAATCCAGCGCGTCGTGATGTCACTTGGTGAATACATGAAGGTTCATTCACATGCTTGC<br>ATTGGTGGTACCAACGTGCGTGAAGATGCCCGCAATTTGGATTCCGGATGCCATGTTGTTGTTGGC<br>ACACCAGGGCGTGTTTACGACATGATTAATCGCAAAGTGCTTCGTACCCATAACATCAAGTTGTTCG<br>TTTTGGATGAAGCCGATGAGATGTTGTCTCGTGGTTTCAAAGATCAAATCCAAGATGTTTTTAAAAT<br>GCTTCCCCCAGACGTTCAGGTCATCTTGCTATCTGCCACTATGCCGCCCGATGTTCTTGAAGTCAG<br>CCGTTGCTTTATGCGTGATCCAGTGAGCATTTTGGTGAAAAAAGAAGAACTTACTTTGGAAGGTATT<br>AAGCAATTTTACGTGAACGTAAAGCAGGAAAACTGGAAGCTTGGTACTTTATGCGATTTGTATGATA<br>CACTTTCAATTACTCAGTCTGTAATTTTCTGTAACACAAGGCGTAAGGTGGATCAGTTGACTCAAGA<br>AATGACAAATCACAACTTTACTGTATCTGCTATGCATGGTGATATGGAGCAACGCGATCGCGAGGTT<br>ATTATGAAGCAATTCCGTTCGGGTTCTTCACGTGTTCTTATTACTACTGATTTGCTCGCTCGAGGTAT<br>TGATGTGCAGCAAGTGTCGCTAGTCATTAACTATGACTTGCCATCGAATAGAGAAAATTACATTCAT<br>AGAATCGGTCGTGGTGGACGGTTTGGTCGCAAAGGTGTTGCTATTAATTTTATAACGGACGAAGAT<br>CGGAGAATTCTTAAAGATATTGAACAATTCTACCATACCACTATTGAAGAAATGCCTGCTAATATTGC<br>CGATTTGATTTAA |
| *A. suspensa eIF4A1*-wt | ATGGATGATCGAAGTGATATAGCTCATGAAGGTCCAGGCGGTATGGATCCTGAGGGTGTCATCGA<br>GTCAACTTGGCACGAGGTGTATGATAATTTCGATGACATGAATTTGCGCGAAGAGTTATTGCGAGG<br>TATCTATGGTTATGGTTTTGAAAAGCCTTCTGCTATTCAGCAGCGTGCTATCATTCCTTGTGTAAAG<br>GGCCGCGACGTTATTGCCCAGGCGCAGTCGGGTACGGAAAAACTGCTACTTTTTCCATTGCAATC<br>TTGCAACAAATCGATACGTCCATCCGTGACTGTCAAGCGTTGATTTTGGCCCCAACTAGGGAATTG |

| | |
|---|---|
| | GCAACCCAAATCCAGCGCGTCGTGATGTCACTTGGTGAATACATGAAGGTTCATTCACATGCTTGC<br>ATTGGTGGTACCAACGTGCGTGAGGATGCCCGCAATTTGGATTCCGGATGTCATGTTGTTGTTGGC<br>ACACCAGGGCGTGTTTACGACATGATTAATCGCAAAGTGCTTCGTACCCATAACATCAAGTTGTTCG<br>TTTTGGATGAAGCCGATGAGATGTTGTCTCGTGGTTTCAAAGATCAAATCCAAGATGTTTTTAAAAT<br>GCTTCCCCCAGACGTTCAGGTCATCTTGCTATCTGCCACTATGCCGCCCGATGTTCTTGAAGTCAG<br>CCGTTGCTTTATGCGTGATCCAGTGAGCATTTTGGTGAAAAAAGAAGAACTTACTTTGGAAGGTATT<br>AAGCAATTTTACGTGAACGTAAAGCAAGAAAACTGGAAGCTTGGTACTTTATGCGATTTGTATGATA<br>CACTTTCAATTACTCAGTCTGTAATTTTCTGTAACACAAGGCGTAAGGTGGATCAGTTGACTCAAGA<br>AATGACAAATCACAACTTTACTGTATCTGCTATGCATGGTGATATGGAGCAACGCGATCGCGAGGTT<br>ATTATGAAGCAATTCCGTTCGGGTTCTTCACGTGTTCTTATTACTACTGATTTGCTCGCTCGAGGTAT<br>TGATGTGCAGCAAGTGTCGCTAGTCATTAACTATGACTTGCCATCGAATAGAGAAAATTACATTCAT<br>AGAATCGGTCGTGGTGGACGGTTTGGTCGCAAAGGTGTTGCTATTAATTTTATAACGGACGAAGAT<br>CGGAGAATTCTTAAAGATATTGAACAATTCTACCATACCACTATTGAAGAAATGCCTGCTAATATTGC<br>CGATTTGATTTAA |

## Supporting Figures:

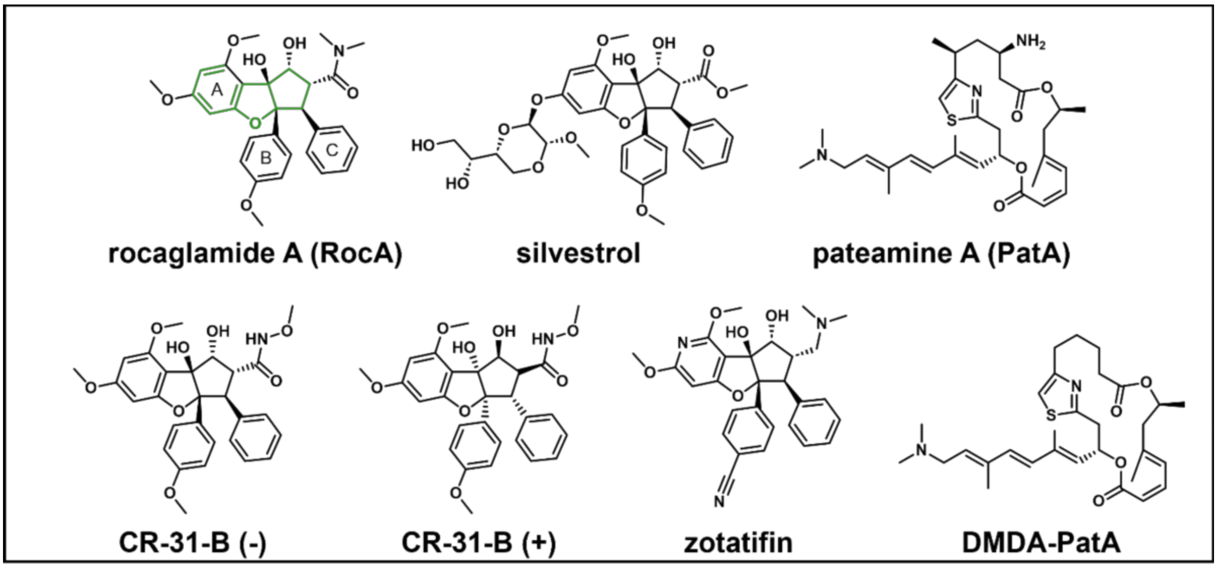


**S1 Fig. Chemical structures of eIF4A inhibitors.** Top: the natural rocaglates rocaglamide A (RocA) and silvestrol isolated from the plant *Aglaia foveolate* and the natural pateamine pateamine A (PatA) isolated from the marine sponge *Mycale hentscheli*. The characteristic cyclopenta[b]benzofuran ring system of rocaglates is highlighted in green and rings A, B and C, which fit into the respective pockets A, B and C in the eIF4A binding pocket are labelled in the RocA structure. Bottom: the synthetic rocaglates CR-31-B (-), CR-31-B (+), and zotatifin, and the synthetic pateamine desmethyl desamino pateamine A (DMDA-PatA).

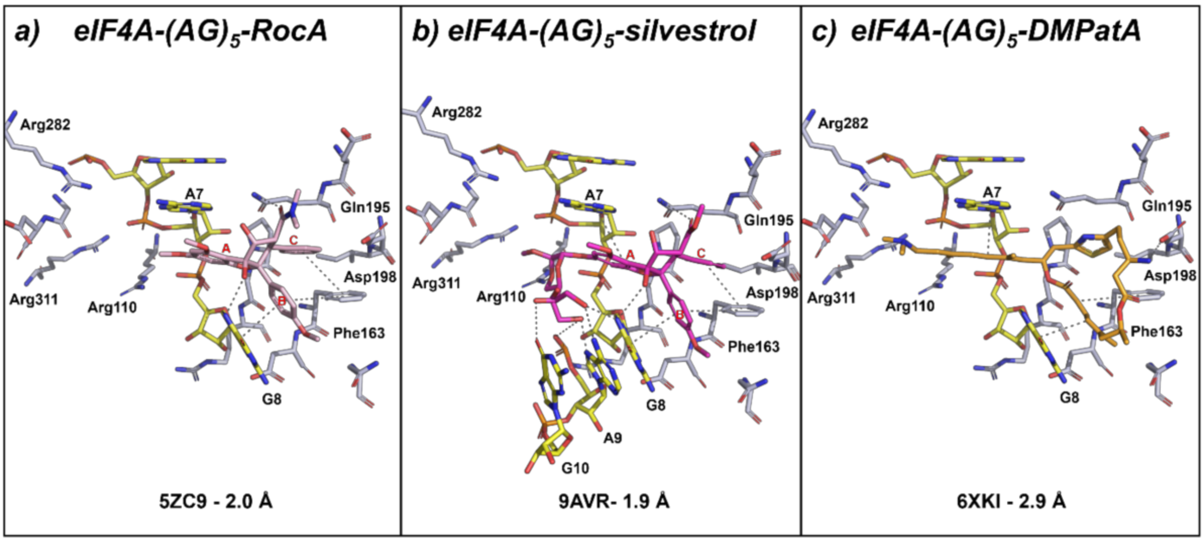


**S2 Fig. Crystal structure comparison of the eIF4A-(AG)5 complex clamping by a) RocA, b) silvestrol and c) DMPatA.** a-b) Phe163 is involved in π-π stacking interaction with rings B and C (marked in red letters) of RocA (a) and silvestrol (b). Ring B is also engaged in additional π-π stacking interactions with RNA G8 and ring A with RNA A7. Both rocaglates (a, b) are further stabilized in the RNA binding pocket by hydrogen-bonds with Gln195 and G8. b) Silvestrol has an additional dioxanyloxy-moiety which further interacts with the RNA residues A9 and G10 as well as with Arg110. c) DMPatA mimics the typical interactions of rocaglates except for the loss of interactions in pocket C. In addition, it is involved in a hydrogen bond with Asp198.

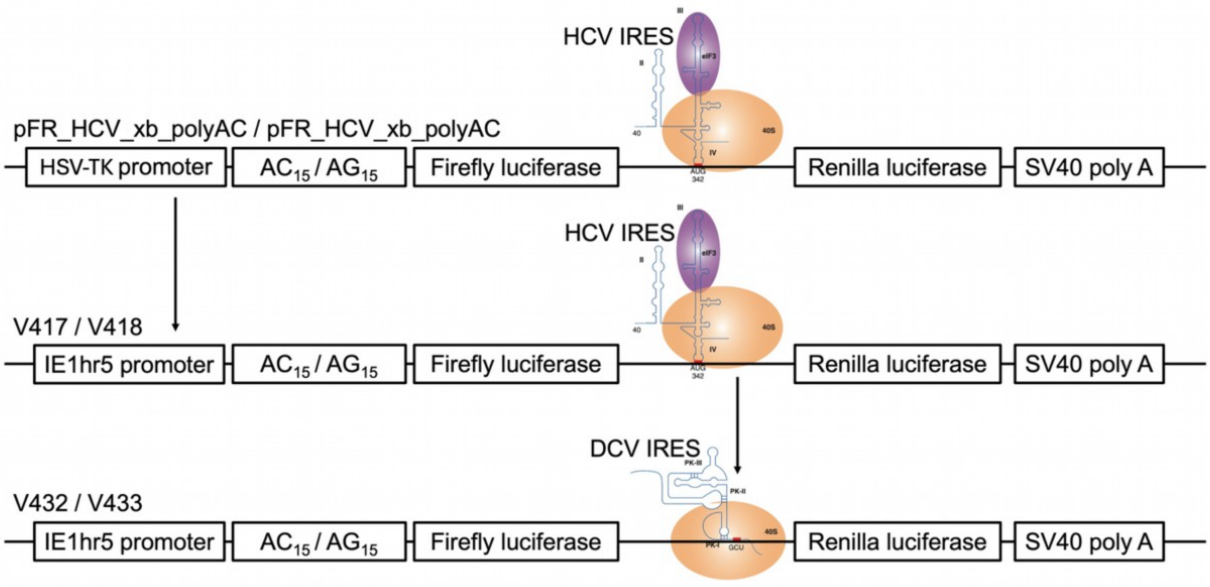


**S3 Fig. Cloning scheme of insect-adapted dual-luciferase vectors** to analyse the effect of silvestrol on translation efficiency in insect cells. In the first step the HSV-TK promoter was replaced with the hr5-ie1 enhancer-promoter. In the second step the Type III Hepatitis C virus (HCV) internal ribosome entry site (IRES) was replaced with a Type IV *Drosophila* C virus IRES. Translation of both IRES types is eIF4A1-independent [1].

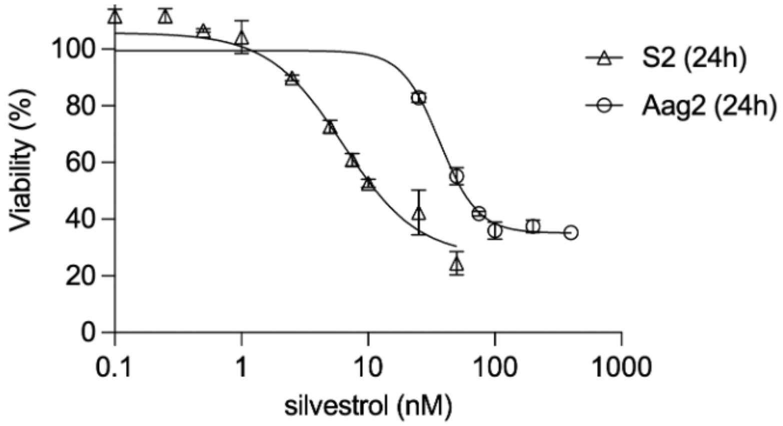


**S4 Fig. Dose-viability curves of silvestrol for $CC_{50}$ value determination** in *D. melanogaster* S2 cells and *Ae. aegypti* Aag2 cells. Incubation time in the presence of silvestrol was 24 h. Data shown for S2 cells are based on 3-7 replicates in 2-3 independent experiments, except for 0.01 nM - 1 nM, which were only conducted with 2 replicates in one experiment. Data shown for Aag2 cells are based on 2 replicates in one experiment, except for 0 and 400 nM, which was only determined once. $R^2$ (S2) = 0.9347, $R^2$ (Aag2) = 0.9861; error bars represent SEM.

**References:**

[1] Svitkin YV, Siddiqui N, Sonenberg N. Protein synthesis initiation in eukaryotes: IRES-mediated internal initiation. eLS. 2015:1–11.
